# Current Advances in the Biochemical and Physiological Aspects of the Treatment of Type 2 Diabetes Mellitus with Thiazolidinediones

**DOI:** 10.1155/2016/7614270

**Published:** 2016-05-23

**Authors:** D. Alemán-González-Duhart, F. Tamay-Cach, S. Álvarez-Almazán, J. E. Mendieta-Wejebe

**Affiliations:** ^1^Laboratorio de Biofísica y Biocatálisis, Sección de Estudios de Posgrado e Investigación, Escuela Superior de Medicina, Instituto Politécnico Nacional, Plan de San Luis y Salvador Díaz Mirón, Casco de Santo Tomás, 11340 Ciudad de México, DF, Mexico; ^2^Laboratorio de Investigación en Bioquímica, Departamento de Formación Básica Disciplinaria y Sección de Estudios de Posgrado e Investigación, Escuela Superior de Medicina, Instituto Politécnico Nacional, Plan de San Luis y Salvador Díaz Mirón, Casco de Santo Tomás, 11340 Ciudad de México, DF, Mexico

## Abstract

The present review summarizes the current advances in the biochemical and physiological aspects in the treatment of type 2 diabetes mellitus (DM2) with thiazolidinediones (TZDs). DM2 is a metabolic disorder characterized by hyperglycemia, triggering the abnormal activation of physiological pathways such as glucose autooxidation, polyol's pathway, formation of advance glycation end (AGE) products, and glycolysis, leading to the overproduction of reactive oxygen species (ROS) and proinflammatory cytokines, which are responsible for the micro- and macrovascular complications of the disease. The treatment of DM2 has been directed toward the reduction of hyperglycemia using different drugs such as insulin sensitizers, as the case of TZDs, which are able to lower blood glucose levels and circulating triglycerides by binding to the nuclear peroxisome proliferator-activated receptor gamma (PPAR*γ*) as full agonists. When TZDs interact with PPAR*γ*, the receptor regulates the transcription of different genes involved in glucose homeostasis, insulin resistance, and adipogenesis. However, TZDs exhibit some adverse effects such as fluid retention, weight gain, hepatotoxicity, plasma-volume expansion, hemodilution, edema, bone fractures, and congestive heart failure, which limits their use in DM2 patients.

## 1. Introduction

The treatment of type 2 diabetes mellitus (DM2) has been directed toward the reduction of hyperglycemia and glycosylated hemoglobin (HbA1c, ≤7%), in order to prevent cardiovascular and other long term risks [1, 2], specially by the usage of insulin sensitizers such as thiazolidinediones (TZDs) [1–5], an effective type of drugs for lowering blood glucose levels as circulating triglycerides [4, 6–9], with adverse effects such as adipocyte differentiation, fluid retention, weight gain, bone loss, and congestive heart failure [6–8, 10–13].

Clinically, pioglitazone is the only available TZD, even though its commercialization has been restricted to a few countries by the US Food and Drug Administration (FDA) since it may cause urinary bladder cancer. The other TZDs, rosiglitazone and troglitazone, show adverse profiles, so they are no longer available in the worldwide market; for example, rosiglitazone was associated with a significant increase in myocardial infarction, heart failure, and death from cardiovascular diseases, so the European Medicines Agency withdrew the approval for this medication in 2010, and the FDA restricted its prescription in the United States [3, 14–17].

In the present review, we summarize the current advances on the biochemical and physiological aspects involved in the treatment of DM2 with TZDs.

**Figure 1:**
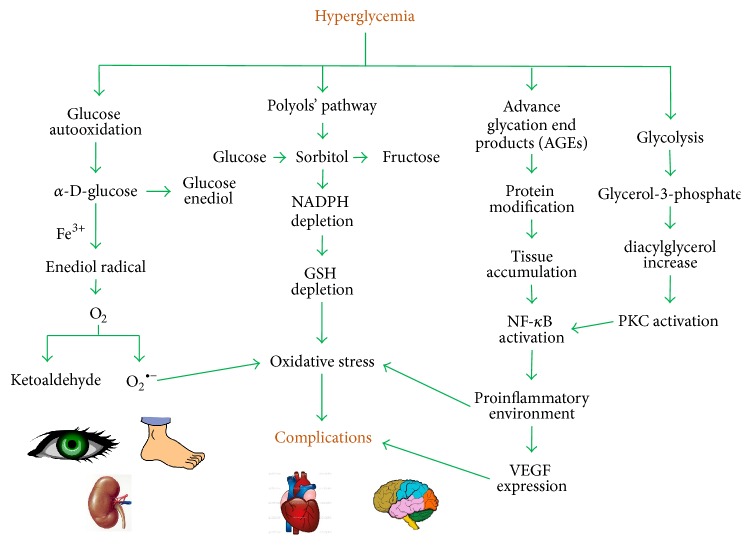
The main pathways triggered by hyperglycemia include glucose autooxidation and constant activation of polyols' pathway and formation of advance glycation end products (AGEs) and excessive glycolysis. With the constant activation of these pathways, living cells and tissues are damaged, mainly by impairment of target protein function, increase in oxidative stress, and activation of signal transduction pathways, leading to the imbalance of normal physiological functions and therefore the development of diabetic complications.

*Type 2 Diabetes Mellitus (DM2)*. DM2 is a metabolic disorder characterized by hyperglycemia, which may be due to a defect in insulin secretion of pancreatic *β* cells, insulin resistance in peripheral tissues, and/or an excessive accumulation of triglycerides and fatty acid derivatives in skeletal muscles. This pathology remains a leading cause of cardiovascular disorders, such as microvascular (retinopathy, nephropathy, and neuropathy) and macrovascular (coronary, cerebrovascular, and peripheral vascular diseases) complications, mainly triggered by the abnormal activation of physiological pathways (Figure 1), and it is also associated with increased risk of cancer, psychiatric illness, cognitive decline, chronic liver disease, and development of arthritis [1–4, 18–24].

The treatment of DM2 is directed toward the reduction of hyperglycemia and HbA1c (≤7%), in order to prevent cardiovascular and other long term risks (Table 1) [1, 2, 5]; there is a wide range of drugs which can be used in order to reduce glycemia, being notable mechanisms such as improving insulin secretion and reducing insulin resistance of peripheral tissues, as the case of TZDs [1–5], which are drugs targeting the peroxisome proliferator-activated receptor gamma (PPAR*γ*).

PPARs have emerged as links between lipids, metabolic diseases, and innate immunity as they regulate energy homeostasis [25, 26], and, specifically talking about PPAR*γ*, this receptor is capable of regulating metabolic genes which will be further discussed and improves insulin sensitivity through glucose and lipid uptake and storage in peripheral tissues such as skeletal muscle, liver, and adipose tissue [26].

The relationship between PPAR*γ* and DM2 has been established using both* in vitro* and* in vivo* experimentation, since it has been seen that the inactivation of PPAR*γ* in mature adipocytes leads to insulin resistance, as mice lacking the receptor develop hyperlipidemia, hyperglycemia, and/or hyperinsulinemia [26, 27].

*Thiazolidinediones (TZDs)*. TZDs are compounds used clinically as insulin sensitizers in order to lower blood glucose levels as circulating triglycerides [4, 6–9], but it has also been shown that these also exhibit other biological activities such as anti-inflammatory, antimalarial, antioxidant, cytotoxic, antimicrobial, and aldose reductase inhibitor activities, either* in vitro* or in animal models [10, 11]. TZDs act as peroxisome proliferator-activated receptors gamma (PPAR*γ*) full agonists, which are also involved in the increase of adipocyte differentiation, fluid retention, weight gain, bone loss, and congestive heart failure. Having such diverse range of pharmacological activities, these molecules have a lot of potential uses, so different strategies have been originated to use them not only for the treatment of DM2 but also for other pathologies [2, 5, 6, 8–11].

When TZDs interact with PPAR*γ*, the receptor regulates the transcription of different genes, mainly those genes involved in glucose homeostasis and adipogenesis, specifically within white adipose tissue (WAT) by inducing brown adipose tissue- (BAT-) like features in it, a unique characteristic exclusive for PPAR*γ* full agonists, such as rosiglitazone [13, 28].

However, despite their excellent potencies, the incidence of undesirable side effects has been linked to the use of TZDs, such as fluid retention, weight gain, hepatotoxicity (only for troglitazone), plasma-volume expansion, hemodilution, edema, and congestive heart failure; it is unknown if the toxicity is mediated by the activation of PPAR*γ* or if it is due to some other mechanism unique to the TZD drug, since neither rosiglitazone nor pioglitazone has displayed the increased incidence of hepatic adverse events seen with troglitazone, suggesting that hepatotoxicity may not be a class effect of PPAR*γ* agonists [6, 7, 29–32]; it has been proposed that the fluid adverse effects may be due to the regulation of PPAR*γ* through an unknown mechanism involved in the enhancement of urinary vasopressin excretion response [33–35].

*Peroxisome Proliferator-Activated Receptors (PPARs)*. PPARs are nuclear receptors that belong to the thyroid/retinoid nuclear family which act as ligand activated transcription factors. Three isoforms for these receptors have been described, *α*, *β*/*δ*, and *γ*, regulating tissue specific target genes involved in biological pathways for lipid and glucose homeostasis. PPAR*α* is expressed predominantly in the liver, heart, and BAT, where it expresses genes involved in fatty acid oxidation; its exogenous ligands are the hypolipidemic fibrate drugs. PPAR *β*/*δ* is expressed in all kinds of tissues and has a crucial role in fatty acid oxidation, mainly in skeletal muscle, liver, and heart. PPAR*γ* is highly expressed in both WAT and BAT, where it functions as a regulator of adipogenesis and as a modulator of lipid metabolism and insulin sensitivity. Activation of PPAR*γ* is crucial for controlling gene networks involved in glucose homeostasis, including increasing the expression of glucose transporter type 4 (GLUT4), adiponectin, resistin, and tumor necrosis factor *α* (TNF*α*), which negatively influence insulin sensitivity. All three isotypes of PPARs, but mainly PPAR*γ*, are ligand activated transcription factors implicated in the physiopathology of various diseases including DM2, obesity, dyslipidemia, atherosclerosis, neoplastic diseases, tumors, inflammatory conditions, and neurodegenerative diseases by forming obligate heterodimers with the retinoid X receptor (RXR), promoting the dissociation of corepressors, recruitment of coactivators, and the subsequent transcription of target genes [3, 6, 12, 13, 36–42].

**Figure 2:**
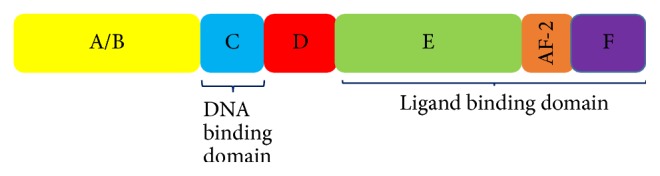
Main functional domains of nuclear PPARs. All three isotypes of PPAR have 4 main functional domains: A/B, which is the activation function 1 (AF-1); C, or DNA binding domain; D, which serves as a hinge between C and E/F; and E/F, which includes AF-2, a ligand binding dimerization transactivation domain.

So far, three isoforms for PPAR*γ* have been described, *γ*1, *γ*2, and *γ*3, which arise as the product of different promoter usage. mRNA of PPAR*γ*1 and PPAR*γ*3 code for the same protein, while PPAR*γ*2 codes for a different protein containing 30 NH_2_-terminal amino acids due to an alternative promoter usage and mRNA splicing, but no physiologically relevant differences in the function of these two isoforms have been determined [9, 29, 37, 38, 43]. PPAR*γ* is organized in main functional domains. The amino terminal A/B domain contains a ligand dependent transactivation function (AF-1), while the C domain is the central DNA binding domain by containing two zinc finger-like structures and one *α* helical DNA binding motif; the E/F domain is the ligand binding domain (LBD), which contains a ligand dependent transactivation function (AF-2), which allows the receptors' conformational changes in the presence of the ligand, leading to the recruitment of coactivators, such as the steroid receptor coactivator type 1 (SRC-1), and the release of corepressors (Figure 2) [12, 44–47].

**Figure 3:**
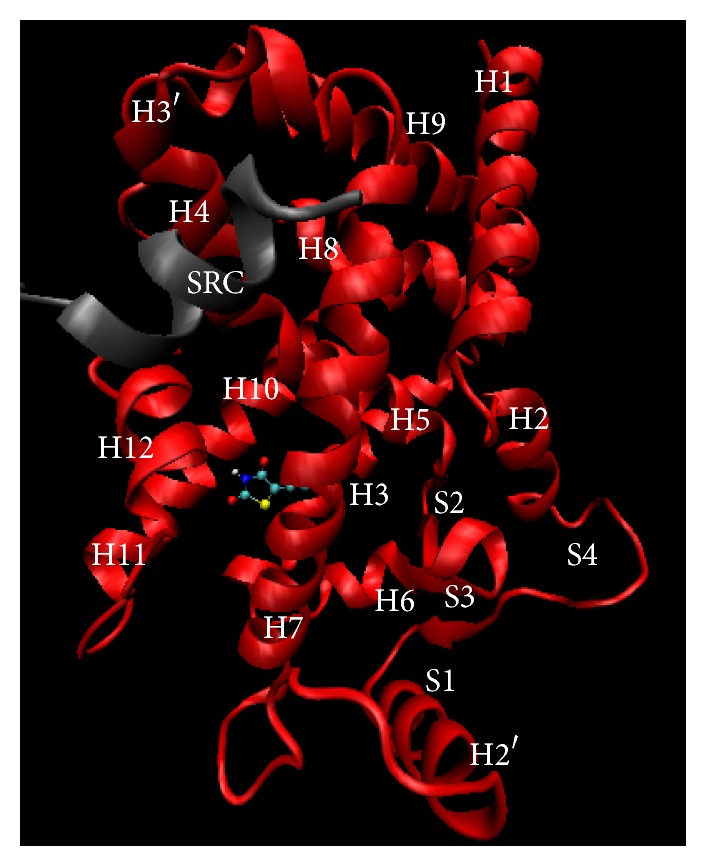
Crystal structure of PPAR*γ* (PDB: 2PRG entry), cocrystalized with rosiglitazone (ligand) and steroid receptor coactivator 1 (SRC-1, coactivator). Figure constructed using Visual Molecular Dynamics (VMD) software.

The PPAR*γ* LBD contains a large binding pocket that allows a wide range of ligands searching for their proper conformations in order to form ligand-receptor complexes. Natural ligands of PPAR*γ* are fatty acids, while synthetic ligands can be classified as either full or partial agonists, such as TZDs, L-tyrosine analogs, and some nonsteroidal anti-inflammatory drugs [7, 8, 12, 30, 31, 39, 44, 48–50]. The structure of the LBD is comprised of 13 helices and 4 *β* sheets, with a total volume of approximately 1300 to 1400 Å. The cavity is Y shaped, consisting of an entrance which extends from the surface of the protein, and then it branches off into two arms, arm I, which extends toward the AF-2 (helix H12), and arm II, situated between helix H3 and the *β* sheet (Figure 3) [44, 45, 47, 51]. An important step during the activation process involves ligand-induced alteration of the conformation of H12 to an active position. The main model for ligand dependent activation of nuclear receptors proposes that agonists stabilize a specific conformation of the AF2 (H12) helix, which, along with helices 3 and 4, provides a suitable interface for binding a coactivator, acting as a molecular switch and creating a binding cleft on the receptor for the coactivator [8, 13, 45].

**Figure 4:**
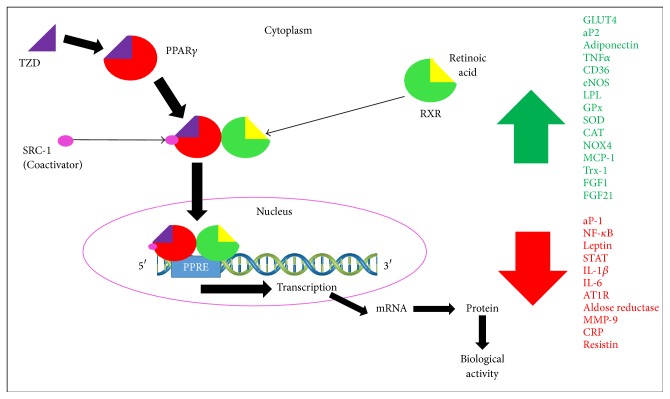
Mechanism of action of PPAR*γ* when it is activated by its exogenous ligands thiazolidinediones (TZDs).

TZDs are one of the most known PPAR*γ* agonists. They share common features such as a hydrophilic head group, a central hydrophobic body, and a flexible linker to a cyclic tail. The hydrophilic head group can have a hydroxyl, carbonyl, or carboxyl oxygen atoms, allowing it to form H bonds with the key amino acid residues Tyr 473, (AF2, H12), His 449 (H11), His323 (H5), Ser289 (H3), and Gln286 (H3) of the LBD, generating an intermolecular network exclusive for full agonists. These H bond networks stabilize the receptor in the proper conformation; however, the acid head group of commercially available TZDs is prone to racemization under physiological conditions due to its stereogenic center at C5, and it has been demonstrated that only the (S)-enantiomers bind to the receptor, which suggests that approximately 50% of the active substance is inactive. Binding of these ligands results in conformational changes of the receptors that facilitate their interaction with coactivator proteins. The resulting complexes activate the transcription of specific target genes, resulting in the induction of signaling cascades that mediate the physiological effects of the ligands (Figure 4) [7–10, 12, 29–31, 39, 43, 44, 47–52].

*PPARγ and Inflammatory Diseases*. Both PPAR*α* and PPAR*γ* isotypes participate in the regulation of inflammation processes. PPAR*α* regulates primarily catabolic and PPAR*γ* regulates primarily anabolic aspects of lipid metabolism [13, 29, 43].

Prostaglandin J2 (PGJ2) activation of PPAR*γ* has been demonstrated to antagonize the activity of activator protein type 1 (aP-1) which enhances the angiogenic response seen in the diabetic microvascular complications [53] and the signal transducer and activator of transcription (STAT) protein which regulates the inflammation cascade [54] and the nuclear factor *κ*B (NF-*κ*B) which also regulates the inflammation cascade mainly in adipocytes [51]; these are known for their positive control on cytokine gene expression [6, 12, 36, 37, 39–41].

Diverse theories propose the molecular mechanisms by which PPAR*γ* exhibits anti-inflammatory effects; among these theories, it can be mentioned that the expression of the receptor is upregulated by oxidized low density lipoproteins (LDL) in macrophages, which will in turn stimulate the expression of the cluster of differentiation 36 (CD36) scavenger receptor gene, resulting in a higher rate of oxidized LDL internalization, which, besides serving as a fatty acid transporter, is a novel biomarker for DM2 [55, 56], but it is also postulated that the expressions of inflammatory mediators such as tumor necrosis factor *α* (TNF*α*), interleukin 6 (IL-6), and matrix metallopeptidase 9 (MMP-9) are negatively controlled by PPAR*γ*, which in turn takes importance for the development of atherosclerosis [50, 57]. It is also possible that PPAR*γ* ligands act as anti-inflammatory and antioxidant agents through the inhibition of the transcription factor NF-*κ*B/p65 and the expression of NADPH oxidase 4 (NOX4), thus reducing the levels of IL-6, C-reactive protein (CRP), and monocyte chemoattractant protein 1 (MCP-1), but it has also been postulated that PPAR*γ* activation directly regulates the expression of endogenous antioxidants such as superoxide dismutase (SOD), catalase (CAT), glutathione peroxidase (GPx), and thioredoxin (Trx-1), therefore playing a crucial role in cardiac redox balance [58–61].

Since diabetic vascular complications are partly mediated by inflammatory processes, the use of TZDs may contribute positively to patients' outcomes since insulin sensitizers suppress the inflammatory processes not only through lowering hyperglycemia but also by modulating the expression of key inflammatory biomarkers as can be seen in Figure 1. These effects may be potentiated when TZDs are used along with other drugs, for example, statins, fibrates, and inhibitors of renin-angiotensin-aldosterone system, which reduce the overall risk for DM2 [62, 63].

It also has been seen that TZDs may exert antiatherogenic effects on vessel wall cells, possibly by downregulating NF-*κ*B inflammatory pathways [62, 63].

*PPARγ and Metabolic Disorders*. PPAR*γ* is predominately expressed in adipose tissue, but it is also expressed in the lungs, placenta, heart, and leukocytes, where it regulates lipid, glucose, and insulin uptake into adipocytes, as it is responsible for regulating the expression of two markers of terminal adipocyte differentiation, adipocyte protein type 2 (aP-2) and phosphoenolpyruvate carboxykinase. PPAR*γ* is also in charge of regulating the expression of the genes which code for lipoprotein lipase (LPL), increasing triglycerides lipolysis in very low density lipoproteins (VLDLs) and increasing high density lipoproteins (HDLs) [62], the fatty acid transport protein, which regulates fatty acid uptake, and fatty acid translocase, which enhances fatty acid uptake in adipocytes, as the repression of the expression of the* ob* gene for leptin, which increases the appetite (Figure 4), and this is concordant with the physiological effects of TZDs, such as lowering blood glucose levels and improving insulin sensitivity [3, 12, 13, 29, 32, 42, 57]. However, adipogenesis caused in response to treatment with TZDs has been linked mainly to the identification of two PPAR*γ*-responsive members of the fibroblast growth factor family, fibroblast growth factor 1 (FGF1) and fibroblast growth factor 21 (FGF21), which act locally in visceral adipose tissue, promoting insulin sensitization, so that the activation of the receptor in the brain, rather than in adipose tissue, has a major role in TZD-induced weight gain [13].

Obesity rates and westernization of lifestyle lead to the increase of dysfunctional adipose tissue, which constantly activates NF-*κ*B and delivers inflammatory cytokines such as TNF*α*, resistin, IL-6, and IL-1*β*, which, along with the impairment of reactive oxygen species (ROS) and water retention, are mainly present in a wide range of diseases like insulin resistance, DM2, hypertension, hyperlipidemia, and cardiovascular diseases (CVD), therefore maintaining a chronic inflammatory environment [1, 14, 58, 64–67].

Insulin resistance has been identified as a major contributor to the development of DM2 and metabolic syndrome since it increases the delivery of fatty acids (FA) into the circulation, which modulate the ability of the heart to use glucose as a source of energy [59, 66, 68–71] leading to a cellular stress characterized by an excessive ROS production, impaired state of nitric oxide (NO) vasorelaxation, production of inflammatory cytokines, mitochondrial dysfunction, increased advanced glycation end products (AGEs), and dysfunction of endothelial progenitor cells, as the inhibition of the antiatherogenic adipokine adiponectin [15, 59, 64, 66, 68–72].

PPAR*γ* is highly expressed in the vascular system, where it is involved in the repression or expression of certain genes such as angiotensin type 1 receptor (AT1R), which can prevent or ameliorate endothelial dysfunction and atherosclerosis [3, 15, 16, 60, 67, 73, 74]. In accordance with this, it has been seen that, in animal models, repression of the expression of PPAR*γ* promotes cardiomyopathy, lipid deposition, arrhythmias, hypertrophy, and increased expression of cardiac inflammatory markers [61, 66, 70, 71]. It has also been shown that adiponectin increases through PPAR-responsive element in the promoter of adipocytes, playing an essential role for the vascular protective effects of PPAR*γ* agonists, as the case where diabetic* db*/*db* mice treated with rosiglitazone stimulated the release of adiponectin, which activated AMP activated protein kinase (AMPK/eNOS) and protein kinase A (cAMP/PKA) pathways in the aorta, consequently leading to the reduction of oxidative stress and the enhancement of NO bioavailability, improving endothelial function [15, 58, 75]. It has also been seen either in clinical practice or in animal models that the continuous treatment with TZDs tends to attenuate the progression of carotid artery intima/media thickness, reducing the onset of restenosis, mainly due to the inhibition of smooth cell migration, the increased apoptosis in vascular smooth cells, and the prevention of insulin driven atherosclerosis by switching myocardial substrate metabolism toward glucose [14, 70, 73, 74, 76].

*PPARγ and Cardiovascular Diseases*. CVD and DM2 are intimately linked as they share some pathophysiological features [76], for example, the development of atherosclerosis, which may lead to myocardial infarction, coronary heart disease, peripheral artery disease, and critical limb ischemia [77].

It has been previously found that CVD increase the rate of cardiovascular death nearly fivefold in subjects with diabetes, mainly due to myocardial infarction. Also, the relevance of diabetes for the development of atherosclerosis has been made clear through the observation that a majority of patients with coronary heart disease have insulin resistance or have been diagnosed with frank diabetes [77, 78].

The use of TZDs has been controversial in terms of prevention of CVD, since it has been shown that these types of drugs induce and maintain the regression of carotid intima-media thickness in patients with type 2 diabetes, as they have been related to anti-inflammatory and antiproliferative activities in smooth muscle cells, inhibiting the atheromatous plaque progression [13, 77]. Another substantial side effect of TZDs is the fluid retention with associated peripheral edema by the alteration of sodium and water reabsorption in the distal collecting ducts of the kidney, which increases the risk for adverse cardiovascular events, such as congestive heart failure [13].

*PPARγ and Bone Fractures*. The use of TZDs has been related to bone fractures, especially rosiglitazone, which exhibited an increased risk of fractures in comparison with patients receiving metformin or glyburide [28]. On the other hand, pioglitazone exhibited an increased incidence of distal extremity fractures on the PRO-active trial [3]. These fractures may be due to the expression of PPAR*γ* in bone narrow stromal cells, osteoblasts, and osteoclasts [28], which promotes an alteration on the mesenchymal stem cell maturation, leading to a shift from an osteoblastic lineage to the adipogenic lineage, which turns into an accumulation of ROS and apoptosis of the cells in the osteogenic lineage, thereby decreasing bone formation [13, 28].

## 2. Conclusions

A great number of studies have shown that the activation of PPAR*γ* is implicated in the development of undesirable adverse effects such as fluid retention, weight gain, hepatotoxicity, plasma-volume expansion, hemodilution, edema, bone fractures, and congestive heart failure, but it is also involved in the prevention of developing atherosclerosis, even though there are certainly another great number of studies which can demonstrate the opposite and also confirm that some agonists of the receptor, specifically rosiglitazone, may increase CVD risk. We believe that the associated risk of CVD during TZDs therapy may be related to different transcription patterns in the PPAR*γ* activation due to different ligands, since troglitazone and pioglitazone do not increase CVD risk, as pioglitazone may cause bladder cancer, or rosiglitazone or troglitazone or hepatotoxicity, which is directly correlated to the use of troglitazone or the other TZDs, as they interact in different ways with the receptor and therefore induce different conformations and different interactions with coactivators/corepressors, as different interactions with the responsive element, therefore triggering the transcription of diverse genes. According to these, it would be important to investigate the different conformations of the receptor in the presence of different ligands, either endogenous or exogenous, so that it would be possible to predict which coactivator or corepressor is more susceptible to be recruited, as the possible biochemical and physiological effects of each one. By doing this prediction, it would be possible to design better ligands derived from TZDs with less adverse effects, and it would also be possible to use them for the treatment of other diseases such as cancer, metabolic syndrome, hypertension, obesity, and even CVD.

## Figures and Tables

**Table 1:** Class of drugs used for the treatment of type 2 diabetes mellitus.

Class	Compounds	Mechanism	Physiological action	Advantages	Disadvantages
Biguanides	Metformin	Activates AMP kinase	↓ hepatic glucose production	Low cost, no weight gain, no hypoglycemia, ↓ CVD events	Gastrointestinal side effects, lactic acidosis, vitamin B12 deficiency

Sulfonylureas	Glyburide Glibenclamide Glipizide Glimepiride	Closes K-ATP channels on *β* cell plasma membranes	↑ insulin secretion	Low cost, ↓ microvascular risk	Hypoglycemia, weight gain

Meglitinides	Repaglinide Nateglinide	Closes K-ATP channels on *β* cell plasma membranes	↑ insulin secretion	↓ postprandial glucose	High cost, hypoglycemia, weight gain, frequent dosing

*α*-Glucosidase inhibitors	Acarbose Miglitol	Inhibits intestinal *α*-glucosidase	Slows intestinal carbohydrate digestion/absorption	Moderate cost, no hypoglycemia, ↓ postprandial glucose ↓ CVD events	Modest HbA1c efficacy, gastrointestinal side effects, frequent dosing

DPP4 inhibitors	Sitagliptin Vildagliptin Saxagliptin Linagliptin	Inhibits DPP4 activity, increasing postprandial incretin GLP-1 concentration	↑ insulin secretion ↓ glucagon secretion	No hypoglycemia	High cost, modest HbA1c efficacy, angioedema, pancreatitis

GLP-1 receptor agonists	Exenatide Liraglutide	Activates GLP-1 receptors	↑ insulin secretion ↓ glucagon secretion ↑ satiety slows gastric emptying	No hypoglycemia, weight loss	High cost, gastrointestinal side effects, acute pancreatitis

Bile acid sequestrants	Colesevelam	Binds bile acids in intestinal tract, increasing hepatic bile acid production	↓ hepatic glucose production ↑ incretin levels	No hypoglycemia ↓ LDL	High cost, modest HbA1c efficacy, constipation ↑ triglycerides

Dopamine 2 agonists	Bromocriptine	Activates dopaminergic receptors	Modulates hypothalamic regulation of metabolism ↑ insulin sensitivity	No hypoglycemia ↓ CVD events	High cost, modest HbA1c efficacy, dizziness, syncope, nausea, fatigue

Thiazolidinediones	Pioglitazone Rosiglitazone	Activates the nuclear transcription factor PPAR*γ*	↑ insulin sensitivity	No hypoglycemia ↑ HDL ↓ triglycerides ↓ CVD events	High cost, weight gain, edema/heart failure, bone fractures, bladder cancer (pioglitazone) ↑ LDL

Insulin	Human NPH Human regular Lispro Aspart Glulisine Glargine Detemir Premixed	Activates insulin receptors	↑ glucose disposal ↓ hepatic glucose production	Universally effective ↓ microvascular risk	Variable cost, hypoglycemia, weight gain
